# Human Herpesvirus 8 Genotype E in Patients with Kaposi Sarcoma, Peru

**DOI:** 10.3201/eid1609.100381

**Published:** 2010-09

**Authors:** Olivier Cassar, Marie-Lise Blondot, Salim Mohanna, Gregory Jouvion, Francisco Bravo, Vicente Maco, Renan Duprez, Michel Huerre, Eduardo Gotuzzo, Antoine Gessain

**Affiliations:** Author affiliations: Institut Pasteur, Paris, France (O. Cassar, M.-L. Blondot, G. Jouvion, R. Duprez, M. Huerre, A. Gessain);; Centre National de la Recherche Scientifique, Paris (O. Cassar, M.-L. Blondot, R. Duprez, A. Gessain);; Universidad Peruana Cayetano Heredia, Lima, Peru (S. Mohanna, F. Bravo, V. Maco, E. Gotuzzo)

**Keywords:** Human herpesvirus 8, HHV-8, Kaposi sarcoma, epidemiology, molecular epidemiology, Peru, viruses, dispatch

## Abstract

To determine human herpesvirus 8 (HHV-8) K1 genotypes in patients with Kaposi sarcoma (KS) from Peru, we characterized HHV-8 in 25 KS biopsy samples. Our findings of 8 A, 1 B, 14 C, and 2 E subtypes showed high HHV-8 diversity in these patients and association between E genotype and KS development.

Human herpesvirus 8 (HHV-8; also known as Kaposi sarcoma–associated herpesvirus) is the etiologic agent of all forms of Kaposi sarcoma (KS) (*1**,**2*). In 2002, the number of KS cases worldwide was ≈65,000, nearly 1% of all diagnosed cancer cases (*3*). KS occurs commonly during HIV-1 infection (AIDS-KS); in transplant recipients; and in persons not infected with HIV, predominantly elderly men of Mediterranean and Middle Eastern origin (classic KS) or in children and adult men from eastern and Central Africa (endemic KS).

Sequence analysis of the highly variable open reading frame (ORF) K1 of HHV-8 has enabled the identification of 5 main HHV-8 molecular subtypes, A–E (*4*). A and C subtypes are prevalent in Europe, Mediterranean countries, the United States, northwestern People’s Republic of China, and southern Siberia; subtype B, in sub-Saharan Africa; and subtype D, in Japan and Oceania. Subtype E is found among Native Americans (*5**–**9*). To our knowledge, KS has been reported in patients infected by all HHV-8 subtypes, except E.

Recent studies demonstrated that classic KS is common in Peru and that AIDS-KS incidence is increasing because of the spread of HIV infection (*10**,**11*). Classic and epidemic KS occurred in patients of Amerindian origin (Quechuas) and in mestizos, reflecting the multiethnic origin of the Peruvian population.

A goal of our study was to determine the HHV-8 genotypes for a series of classic KS or AIDS-KS cases in Peru. We also aimed to report KS in patients infected by an E subtype.

## The Study

We studied a series of 36 KS tumors diagnosed during 1989–2002 at the Hospital Nacional Cayetano Heredia in Lima. All these formalin-fixed, paraffin-embedded biopsy samples were stained by using hematoxylin–eosin stain and Perl methods. Immunohistochemistry was performed on deparaffinized sections by using monoclonal antibodies directed against CD34 and latent nuclear antigen (LANA-1) (*12*).

DNA was extracted from paraffin blocks by using the QIAamp DNA Mini Kit (QIAGEN GmbH, Hilden, Germany). HHV-8 infection was determined by nested PCR to obtain a 220-bp (variable region [VR] 1–inner fragment) and a 240–300-bp (VR2-inner) fragment of the ORF-K1 (*13*). Phylogenetic trees were generated with the neighbor-joining method (PAUP* version 4.0b10; http://paup.csit.fsu.edu) on fragments of either 309 bp (VR1-outer) or 165 bp (VR1-inner) of the VR1 (K1 gene) by using different sequence prototypes of the 4 major HHV-8 genotypes (*13**,**14*).

**Figure 1:**
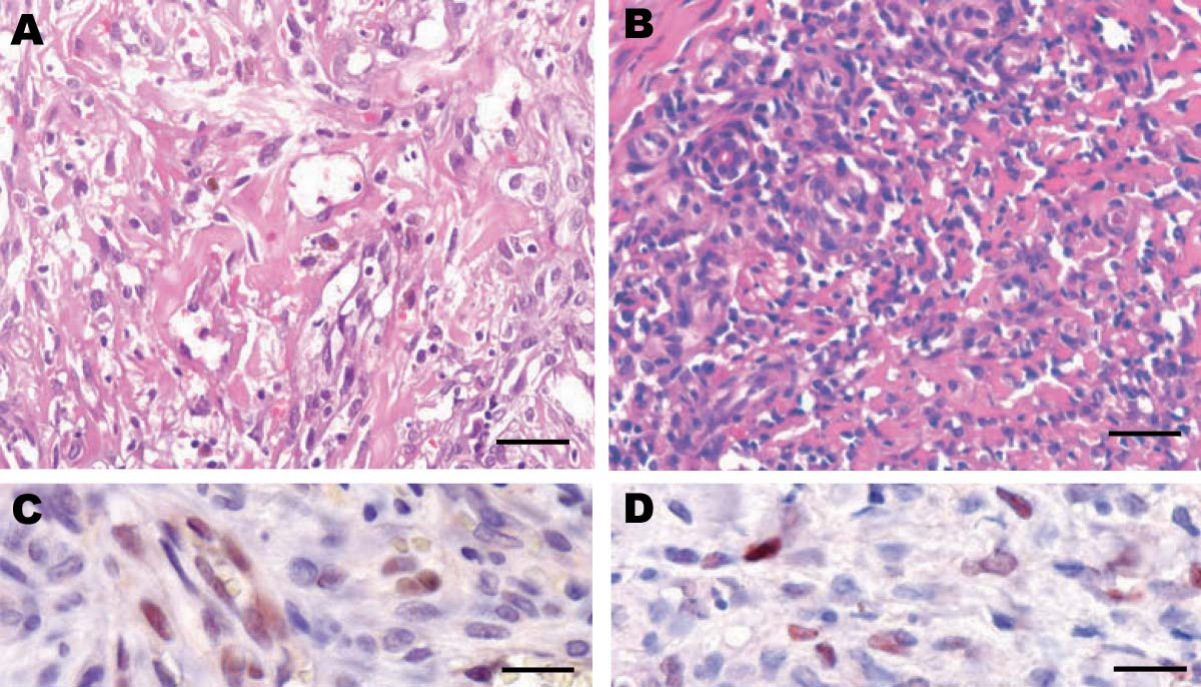
Histologic patterns of cutaneous Kaposi sarcoma (KS) associated with a human herpesvirus 8 (HHV-8) type E infection. Patient 1: A) The spindle cells were organized as bundles, forming vascular slit-like spaces containing erythrocytes. Some macrophages containing hemosiderin were observed (data not shown). Scale bar = 25 μm. C) Immunohistochemical testing showed a positive signal for HHV-8 infection (latent nuclear antigen [LANA-1]) and CD34 (data not shown). The Perls staining also gave highly positive results (data not shown). Scale bar = 50 μm. (Patient 1 corresponds to the first patient [04/0480] in the Table A1], a 51-year-old mestizo man who had HIV-1 infection.) Patient 2: B) Spindle cells forming rare vascular channels, with numerous lymphocytes, plasma cells, and macrophages. Scale bar = 25 μm. D) Immunohistochemistry showed a lower positive signal for HHV-8 infection (LANA-1) and CD34 (data not shown). Few cells displayed a positive Perls staining (data not shown). Scale bar = 50 μm. (Patient 2 corresponds to the tenth patient [06/0772] in the online Table A1, a 24-year-old mestizo man with HIV-1 infection).

Histopathologic analysis was originally conducted on 36 biopsy samples, mostly from skin, diagnosed as KS. Three patterns were observed by hematoxylin–eosin stained specimens. The first was characterized by dilated, irregular, and angulated blood vessels in the dermis, associated with a variable number of lymphocytes. In the second pattern, dermal vascular channels lined by plump spindle cells were seen; some of these spindle cells coalesced to form aggregates, which were poorly delineated and often located around blood vessels. The third pattern (nearly half of all biopsy samples) was characterized by well-delineated sheets and bundles of spindle cells, which coalesced to form nodules. The proportion of spindle cells labeled with the monoclonal antibody LANA-1 varied according to the histopathologic pattern; the bundles and sheets of spindle cells in the third pattern displayed the strongest signal (Figure 1, panels A and B).

DNA was extracted from the 36 formalin-fixed, paraffin-embedded biopsy samples; DNA quantity and quality were appropriate in 30 biopsy samples. A faint PCR signal of the expected size was seen after single PCR in 12 samples for VR1 (VR1-outer) and in 4 cases for VR2 (VR2-outer) amplification. After nested PCR, a signal was obtained in 25 cases for VR1-inner and in 17 cases for VR2-inner (Table A1). After cloning and sequencing procedures, the HHV-8 genotype was obtained for 25 different KS cases, of which 8 genotypes belonged to the A subtype, including an A5, and 14 belonged to the C subtype. An E subtype was identified for 2 patients. In 1 case, a B subtype was determined. Among these 25 sequences, 16 were unique and 9 formed 3 groups of identical sequences. The 25 VR1-inner sequences exhibited 0%–24.4% nt divergence and 0%–43.1% aa acid divergence among pairwise comparisons. When the VR1-outer and VR2-inner sequences (536 bp) of the 2 E subtype strains in Peru were combined, the nucleotide divergence was ≈7% and reached 10% at the amino acid level. The 04480 and 06772 E subtype strains were closer to the Brazilian Amerindian strains (Kat, Sio, Wai, Tir, Tupi) than to the Ecuadorian (Hua1, Hua2, Hua3) or French Guianan Amerindian (Wagu) strains.

**Figure 2:**
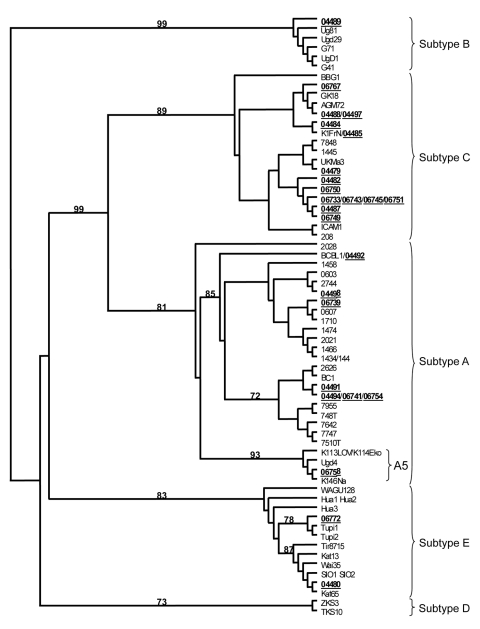
Unrooted phylogenetic tree generated with the neighbor-joining method (PAUP* version 4.0b10; http://paup.csit.fsu.edu) on the best 165-bp alignment of the variable region [VR] 1 comprising 79 human herpesvirus 8 nt sequences, including 25 novel sequences generated (GenBank accession nos. GU827339–GU827363). The strains were aligned with Data Analysis in Molecular Biology software (http://dambe.bio.uottawa.ca/software.asp), and the final alignment was submitted to the ModelTest software version 3.6 (http://darwin.uvigo.es/software/modeltest.html) to select, according to the Akaike Information Criterion, the best model to apply to phylogenetic analyses. The selected model was the general time reversible model. The reliability of the inferred tree was evaluated by bootstrap analysis on 1,000 replicates. Bootstrap support is noted on the branches of the tree.

Phylogenetic analyses were performed by using 2 sets of sequences; 25 VR1-inner (Figure 2) and 11 VR1-outer sequences (data not shown). Forty-seven prototype strain sequences were added. The main molecular HHV-8 subtypes, A–E, were identified on the basis of consistent topology and bootstrap values obtained (Figure 2; data are not shown for other phylogenetic analyses performed, for example with the 11 VR1-outer sequences obtained after the first round of PCR). Among the 25 VR1-inner sequences, 22/25 were located in the large A/C subtype, and 8 strains belong to the A subtype, with strains scattered among 3 different subgroups (Figure 2). The 06758 strain belongs to the typical sub-Saharan A5 group. Fourteen strains are distributed among different groups in the C subtype, and the remaining sequences clustered in the sub-Saharan African B (04489) and Amerindian E (06772 and 04480) subtypes.

Two AIDS-KS mestizo patients (Figure 1) were thus found to be infected by typical E subtype HHV-8 strains: a 51-year-old man with a tumor on his neck and a 24-year-old man with multiple tumors on the upper limbs. These tumors were mostly macroscopic nodules but displayed major histologic differences (Figure 1). CD34 (data not shown) and LANA-1–positive cells (Figure 1, panels C and D) were noted in both KS biopsy samples.

## Conclusions

HHV-8 K1 gene characterization of KS tumor biopsy samples demonstrated high molecular genotype diversity, including 4 of the 5 main known molecular subtypes. The most frequent were the A and C subtypes, typically of European origin. Other patients were infected by a sub-Saharan African HHV-8 genotype (1 A5 and 1 B) or by HHV-8 genotype E strains of Amerindian origin.

Such findings were not unexpected because persons in Peru are of many ethnicities. Indeed, the genetic background of the Peruvian population is diverse, reflecting the multiple waves or populations that colonized the country during the last millennium. Schematically, this began with different Amerindians groups in the Lithic period (infected possibly with E subtype), followed by the Spanish colonization of the Americas mainly from southern Europe (infected possibly by A and C subtypes) and later slave trade from Africa (infected by A5 or B subtypes). Such HHV-8 strain diversity has been previously observed in French Guiana (*6**,**14*).

HHV-8 genotype E previously has been found exclusively in Amerindians from Central and South America, including Brazil (*5**,**9*), Ecuador (*7*), and French Guiana (*6*). In each instance, HHV-8 was detected in blood samples, and there was some debate about the presence/development of KS in such highly infected populations (*15*).

We demonstrated that KS can occur in HHV-8 subtype E–infected persons. Indeed, in 2 AIDS-KS patients, an E genotype was characterized in the tumor lesions. Further studies are ongoing to provide new insights into the distribution and genetic epidemiology of such HHV-8 infection in Amerindian populations.

## Appendix

**Table A1:** Patient and tumor characteristics and results of immunohistochemical analyses and molecular typing of VR1 and VR2 of the open reading frame K1 of HHV-8 in patients with KS, Peru*

ID IP	Age, y/sex	Origin	Lesion	No. lesions	Biopsy site	HIV status	Clinical diagnosis	Level of spindle cells infiltration	Perls	CD 34	LANA-1	VR1 PCR†			VR2 PCR		Subtype by molecular analysis
												VR1 outer	VR1 inner		VR2 outer	VR2 inner	
04/0480	51/M	Mestizo	Nodule	1	Neck	+	AIDS KS	++	+++	++	++	+ E	+ E		–	+ E	E
04/0484	29/M	Mestizo	Nodule	Multiple	Nose	+	AIDS KS	+++	++	++	++	+ C	+ C		–	–	C
04/0485	29/M	Mestizo	Nodule	Multiple	Head	+	AIDS KS	+++	+	++	++	+ C	+ C		–	+ C	C
04/0489	32/M	Mestizo	Macula	1	Hard palate	+	AIDS KS	++	++	++	+	–	+ B		–	+ B	B
04/0492	34/F	Mestizo	Macula	Multiple	Face	+	AIDS KS	+++	+++	++	++	+ A	+ A		–	+ A	A
04/0497	32/M	Mestizo	Patch	Multiple	Chest	+	AIDS KS	+++	+++	++	++	–	+ C		–	+‡	C
06/0749	35/F	Mestizo	Macula	Multiple	Hard palate	+	AIDS KS	+	+	+	+	+ C	+ C		–	–	C
06/0750	35/M	Mestizo	Macula, nodule	Multiple	Hard palate	+	AIDS KS	++	++	+	++	–	+‡ C		–	–	C
06/0758	37/M	Mestizo	Nodule	1	Eyelid	+	AIDS KS	+	++	++	+/−	–	+ A		–	–	A
06/0772	24/M	Mestizo	Papula, nodule	Multiple	Upper limb	+	AIDS KS	+	+	+	+	+ E	+ E		+ E	+ E	E
04/0479	83/F	Mestizo	Papula	1	Lower limb	–	Classic KS	+++	+	+	++	–	+ C		–	+ C	C
04/0482	75/M	Mestizo	Nodule	Multiple	Hand	–	Classic KS	+++	+++	++	++	+‡	+ C		–	+ C	C
04/0487	70/F	Quechua	Nodule	1	Foot	–	Classic KS	+++	+++	+	++	–	+ C		–	+ C	C
04/0488	75/M	Mestizo	Nodule	Multiple	Foot	–	Classic KS	+++	++	+	++	–	+ C		–	+ C	C
04/0498	75/M	Quechua	Nodule	1	Foot	–	Classic KS	+++	+++	++	++	+ A	+ A		+ A	+ A	A
06/0733	58/M	Mestizo	Macula	Multiple	Hard palate	–	Classic KS	–	-	+	+/−	–	+ C		–	–	C
06/0739	46/M	Mestizo	Patch	Multiple	Upper limb	–	Classic KS	+	+/−	++	++	–	+ A		–	+ A	A
06/0741	30/M	Mestizo	Nodule	1	Face	–	Classic KS	+++	+	++	+++	+ A	+ A		+ A	+‡	A
06/0743	30/M	Mestizo	Nodule	Multiple	Face	–	Classic KS	++	++	+	++	+ C	+ C		–	–	C
06/0745	31/M	Mestizo	Papula, nodule	Multiple	Lower limb	–	Classic KS	+	+	+	+	–	+ C		–	–	C
06/0751	26/M	Quechua	Macula	1	Hard palate	–	Classic KS	+++	+++	+	++	+ C	+ C		–	+‡	C
06/0754	63/F	Mestizo	Nodule	Multiple	Neck	–	Classic KS	++	+/−	+++	+++	+ A	+ A		–	+‡	A
06/0767	76/F	Quechua	Nodule	1	Lower limb	–	Classic KS	–	–	+++	–	–	+ C		+ C	+ C	C
04/0491	63/F	Mestizo	Nodule	ND	Foot	–	ND	++	+	+	++	–	+‡ A		–	+‡ A	A
04/0494	75/F	ND	Nodule	ND	Foot	–	ND	++	++	+	++	+ A	+ A		–	–	A

*KS, Kaposi sarcoma; VR1 and VR2, variable region 1 or 2 of the open reading frame K1 of HHV-8; HHV-8, human herpesvirus 8; ID IP, identification of the specimen given by the Institut Pasteur pathologic unit; LANA, latency-associated nuclear antigen; AIDS KS, KS occurring with HIV-1 infection; –, no amplification product. ND, not determined. †For VR1, the first PCR was performed with the primer set VR1S (ATCCTTGCCAAYATCCTGGTATTGBAA) / VR1AS1 (ACGATTTGACAGGCGAGACGACAGC) (amplification of 373 bp) and followed by a nested PCR with a second set of primers VR1S/VR1AS2 (ACAATRCAAAGTAACABGCTGRCC) for the amplification of a 220-bp fragment. For VR2 amplification, the first PCR was performed by using the primer set VR2S (TCTCGCCTGTCAAATCBTMTATGT) / VR2AS1(AGTACCAMTCCACTGGTTGYGTAT) amplification of 314 bp and followed by a nested PCR with a second set of primers VR2S/VR2AS2 (AGTTCCTAMGATACCAMACATGGTT) for the amplification of a 240–300-bp fragment. Amplified PCR products of the appropriate size were then purified from gel, cloned, sequenced as previously described (*14*). Sequences were verified on both DNA strands. ‡Weak PCR signal.
